# Pneumococcal Vaccination Among Medicare Beneficiaries Occurring After the Advisory Committee on Immunization Practices Recommendation for Routine Use Of 13-Valent Pneumococcal Conjugate Vaccine and 23-Valent Pneumococcal Polysaccharide Vaccine for Adults Aged ≥65 Years

**DOI:** 10.15585/mmwr.mm6627a4

**Published:** 2017-07-14

**Authors:** Carla L. Black, Walter W. Williams, Rob Warnock, Tamara Pilishvili, David Kim, Jeffrey A. Kelman

**Affiliations:** ^1^Immunization Services Division, National Center for Immunization and Respiratory Diseases, CDC; ^2^Acumen, LLC, Burlingame, California; ^3^Division of Bacterial Diseases, National Center for Immunization and Respiratory Diseases, CDC; ^4^Center for Medicare, Centers for Medicare & Medicaid Services.

On September 19, 2014, CDC published the Advisory Committee on Immunization Practices (ACIP) recommendation for the routine use of 13-valent pneumococcal conjugate vaccine (PCV13) among adults aged ≥65 years, to be used in series with 23-valent pneumococcal polysaccharide vaccine (PPSV23) (*1*). This replaced the previous recommendation that adults aged ≥65 years should be vaccinated with a single dose of PPSV23. As a proxy for estimating PCV13 and PPSV23 vaccination coverage among adults aged ≥65 years before and after implementation of these revised recommendations, CDC analyzed claims for vaccination submitted for reimbursement to the Centers for Medicare & Medicaid Services (CMS). Claims from any time during a beneficiary’s enrollment in Medicare Parts A (hospital insurance) and B (medical insurance) since reaching age 65 years were assessed among beneficiaries continuously enrolled in Medicare Parts A and B during annual periods from September 19, 2009, through September 18, 2016. By September 18, 2016, 43.2% of Medicare beneficiaries aged ≥65 years had claims for at least 1 dose of PPSV23 (regardless of PCV13 status), 31.5% had claims for at least 1 dose of PCV13 (regardless of PPSV23 status), and 18.3% had claims for at least 1 dose each of PCV13 and PPSV23. Claims for either type of pneumococcal vaccine were highest among beneficiaries who were older, white, or with chronic and immunocompromising medical conditions than among healthy adults. Implementation of the National Vaccine Advisory Committee’s standards for adult immunization practice to assess vaccination status at every patient encounter, recommend needed vaccines, and administer vaccination or refer to a vaccinating provider might help increase pneumococcal vaccination coverage and reduce the risk for pneumonia and invasive pneumococcal disease among older adults (*2*).

CDC monitored PCV13 and PPSV23 claims submitted for reimbursement to CMS among beneficiaries aged ≥65 years who were continuously enrolled in Medicare Parts A and B* during annual periods from September 19, 2009, through September 18, 2016. Enrollment periods covered the 5 years before through 2 years after the recommendation for routine use of PCV13 and PPSV23 in series for adults aged ≥65 years (*1*). The number of beneficiaries per annual enrollment period ranged from 23.7 million to 25.0 million during these years. Beneficiaries were considered to be vaccinated with either PPSV23 or PCV13 or both if a claim for vaccination was submitted at any time during a beneficiary’s history of enrollment in Medicare Parts A and B since reaching age 65 years and before the end of the enrollment period of interest. However, claims are only available in the CMS database beginning January 1, 1999. PCV13 and PPSV23 were identified by current procedural technology codes 90670 and 90732, respectively. Claims submitted from any hospital or outpatient setting (including pharmacies) were included. Claims submitted to CMS for at least 1 PCV13 dose (regardless of PPSV23 status), at least 1 PPSV23 dose (regardless of PCV13 status), at least 1 dose each of PCV13 and PPSV23, and at least 1 dose of either vaccine were stratified by age, race/ethnicity, state of residence, and the presence of chronic or immunocompromising medical conditions for which PCV13 or PPSV23 or both are indicated among adults aged <65 years (*3*). Race/ethnicity was categorized as Hispanic or Latino, black, white, Asian, American Indian/Alaskan Native, and “other.”^†^ Chronic and immunocompromising medical conditions were identified by the presence of *International Classification of Diseases, Ninth Revision *(ICD-9) and ICD-10 codes listed on any claim submitted to CMS during a beneficiary’s history of enrollment in Medicare Parts A and B through the end of the enrollment period of interest. The proportion of beneficiaries with claims submitted for PCV13 by the end of each month during September 2014–September 2016 was also assessed. The denominator for each month included beneficiaries continuously enrolled in Medicare Parts A and B for at least 12 months before and including the month of interest.

By September 18, 2015, 14.8% of Medicare beneficiaries aged ≥65 years had claims for PCV13, and 8.7% had claims for both PCV13 and PPSV23 (Figure 1). By September 18, 2016, claims for PCV13 and claims for both PCV13 and PPSV23 increased to 31.5% and 18.3%, respectively. Claims for PPSV23 increased from 40.0% by September 18, 2010 to 44.5% by September 18, 2014; claims for at least one pneumococcal vaccine of any type increased from 40.0% by September 18, 2010 to 56.4% by September 18, 2016.

**FIGURE 1:**
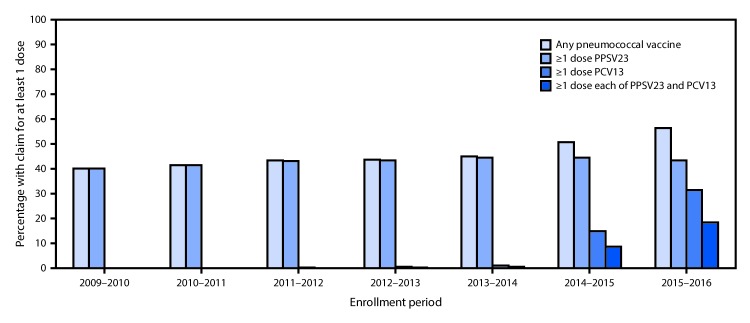
Percentage of Medicare beneficiaries aged ≥65 years with claims submitted for pneumococcal vaccination* — United States, September 2009–September 2016† **Abbreviations:** PCV13 = 13-valent pneumococcal conjugate vaccine; PPSV23 = 23-valent pneumococcal polysaccharide vaccine. * Percentage with at least one claim for pneumococcal vaccination since January 1, 1999 through the end of the enrollment period. ^†^ Each enrollment period extends from September 19 of the first year through September 18 of the subsequent year, with the exception of the 2011–2012 period, which ends on October 12, 2012, corresponding to the date of publication of the first recommendation for the use of PCV13 in series with PPSV23 in adults with certain immunocompromising conditions; denominators include all beneficiaries continuously enrolled in Medicare Parts A and B for the duration of the enrollment period.

Claims for pneumococcal vaccination by September 18, 2016, varied by demographic characteristics and the presence of chronic and immunocompromising medical conditions (Table). The percentages of beneficiaries with claims for all pneumococcal vaccine outcomes were lowest among beneficiaries aged 65–69 years and highest among beneficiaries aged 80–84 years. Claims for PPSV23 were 133% higher among beneficiaries aged 80–84 years (58.5%) than among those aged 65–69 years (25.1%); claims for PCV13 were 33% higher among beneficiaries aged 80–84 years (34.0%) than among those aged 65–69 years (28.2%). Claims for PPSV23, PCV13, or both vaccines were higher among white beneficiaries than among beneficiaries of other racial/ethnic groups; the largest differences were between white and Hispanic beneficiaries (44.6% compared with 32.2% [PPSV23]; 33.1% compared with 13.9% [PCV13]; and 19.5% compared with 6.8% [both PPSV23 and PCV13]). The percentages of beneficiaries aged ≥65 years with chronic medical conditions with claims for PPSV23 (47.1%), PCV13 (33.3%), and both vaccines (19.9%) were higher than for beneficiaries without these conditions (22.2%, 21.8%, and 9.8%, respectively). Similarly, the percentages of beneficiaries with immunocompromising medical conditions with claims for PPSV23, PCV13, and both vaccines were higher than the percentage among beneficiaries without these conditions (50.7% compared with 29.9%, 35.1% compared with 25.2%, and 21.8% compared with 12.2%, respectively).

**TABLE:** Percentage of Medicare beneficiaries aged ≥65 years with claims submitted for pneumococcal vaccination, by age, race/ethnicity, presence of chronic and immunocompromising medical conditions, and state — United States, September 2016*

Characteristic	Total no. enrolled beneficiaries	%			
		≥1 dose PPSV23^†^	≥1 dose PCV13^§^	Both PPSV23 and PCV13^¶^	Any pneumococcal**
**Age group (yrs)**					
65–69	7,939,433	25.1	28.2	11.0	42.2
70–74	6,056,516	43.0	33.2	19.3	56.9
75–79	4,481,971	53.7	34.4	23.7	64.5
80–84	3,179,177	58.5	34.0	24.3	68.2
≥85	3,345,213	57.8	30.1	20.9	67.1
**Race/Ethnicity^††^**					
White	21,436,465	44.6	33.1	19.5	58.3
Black	1,846,978	33.2	19.4	10.3	42.4
Asian	468,070	42.2	23.9	13.1	53.0
Hispanic	379,943	32.2	13.9	6.8	39.3
American Indian/Alaskan Native	113,646	36.5	25.6	12.0	50.1
Other race	423,720	39.5	28.1	15.7	52.0
**Immunocompromising condition^§§^**					
Yes	15,972,169	50.7	35.1	21.8	64.0
No	9,030,141	29.9	25.2	12.2	42.9
**Chronic medical condition^¶¶^**					
Yes	21,104,617	47.1	33.3	19.9	60.5
No	3,897,693	22.2	21.8	9.8	34.2
**State of residence**					
Alabama	468,852	41.5	23.1	13.3	51.3
Alaska	59,323	25.4	23.0	9.4	39.0
Arizona	499,658	41.4	29.9	16.5	54.8
Arkansas	317,732	42.7	27.1	16.1	53.7
California	2,107,110	40.2	27.3	15.1	52.4
Colorado	332,022	43.4	36.9	20.8	59.5
Connecticut	318,829	46.2	34.9	20.5	60.7
Delaware	122,037	48.6	40.5	24.3	64.8
District of Columbia	43,433	38.0	26.8	14.9	49.9
Florida	1,712,605	44.3	26.1	15.8	54.7
Georgia	674,242	42.5	29.9	17.3	55.1
Hawaii	83,703	42.1	33.3	19.7	55.6
Idaho	131,744	38.1	26.2	14.5	49.8
Illinois	1,124,884	43.2	31.1	18.5	55.7
Indiana	592,448	47.6	33.5	20.5	60.5
Iowa	352,685	43.8	41.1	23.7	61.2
Kansas	302,081	41.6	33.0	19.1	55.5
Kentucky	389,989	42.3	26.4	15.5	53.1
Louisiana	348,808	42.5	21.5	12.5	51.4
Maine	150,171	41.2	39.4	22.3	58.4
Maryland	586,357	43.7	36.5	20.9	59.2
Massachusetts	632,551	41.1	43.1	21.1	63.0
Michigan	827,012	45.3	28.9	17.3	56.9
Minnesota	230,895	49.7	47.7	30.3	67.1
Mississippi	314,296	39.1	20.7	11.6	48.2
Missouri	528,914	43.4	32.5	19.6	56.3
Montana	118,967	37.7	36.4	19.7	54.4
Nebraska	205,497	44.8	36.9	22.5	59.1
Nevada	197,861	34.0	23.6	11.8	45.8
New Hampshire	170,816	46.1	47.1	26.9	66.3
New Jersey	889,614	43.0	26.9	15.4	54.5
New Mexico	163,879	40.3	24.1	13.5	50.9
New York	1,332,798	42.3	28.9	17.1	54.1
North Carolina	840,134	46.6	38.6	23.3	61.9
North Dakota	70,805	42.3	41.3	24.0	59.6
Ohio	809,510	46.6	34.3	21.0	59.9
Oklahoma	385,745	44.0	26.8	15.6	55.3
Oregon	275,942	40.8	34.9	19.6	56.2
Pennsylvania	992,016	45.1	41.2	23.9	62.4
Rhode Island	77,264	35.6	31.0	14.1	52.6
South Carolina	507,588	42.6	32.3	18.1	56.7
South Dakota	90,734	41.5	35.4	20.5	56.4
Tennessee	538,577	44.4	32.3	18.8	57.9
Texas	1,661,198	44.3	23.9	14.2	54.0
Utah	154,746	41.0	32.7	16.5	57.2
Vermont	86,650	38.6	37.1	19.8	55.9
Virginia	789,897	46.8	40.1	23.9	63.0
Washington	568,297	40.3	34.3	19.0	55.6
West Virginia	191,465	38.0	21.2	11.5	47.7
Wisconsin	435,666	52.3	53.9	34.9	71.3
Wyoming	70,165	34.8	27.3	13.7	48.4
**Median**	—	42.5	32.5	18.8	55.7
**Range across states**	—	25.4–52.3	20.7–53.9	9.4–34.9	39.0–71.3

**Abbreviations:** PCV13 = 13-valent pneumococcal conjugate vaccine; PPSV23 = 23-valent pneumococcal polysaccharide vaccine.

* Denominator in each subgroup includes all beneficiaries continuously enrolled in Medicare Parts A and B during September 19, 2015–September 18, 2016.

^†^ Percentage of beneficiaries with at least one claim for PPSV23 during January 1, 1999–September 18, 2016.

^§^ Percentage of beneficiaries with at least one claim for PCV13 during January 1, 1999–September 18, 2016.

^¶^ Percentage of beneficiaries with at least one claim for PPSV23 and at least one claim for PCV13 during January 1, 1999–September 18, 2016.

** Percentage of beneficiaries with at least one claim for PPSV23 or PCV13 during January 1, 1999–September 18, 2016.

^††^ Race/ethnicity was categorized as Hispanic or Latino, black, white, Asian, American Indian/Alaskan Native, and “other.” Beneficiaries identified as Hispanic or Latino might be of any race. Beneficiaries identified as black, white, Asian, American Indian/Alaskan Native, or other race are non-Hispanic. Other includes persons of multiple race.

^§§^ Includes cerebrospinal fluid leak; cochlear implant; sickle cell disease or other hemaglobinopathy; congenital or acquired asplenia; congenital or acquired immunodeficiency (including B- or T-lymphocyte deficiency, complement deficiencies, and phagocytic disorders excluding chronic granulomatous disease); HIV infection; chronic renal failure; nephrotic syndrome; leukemia; lymphoma; Hodgkin disease; generalized malignancy; immunosuppression caused by treatment with immunosuppressive drugs, including long-term corticosteroids and radiation therapy; solid organ transplant; and multiple myeloma. Use of *International Classification of Diseases* codes might be nonspecific in identifying generalized malignancies if providers use these codes to rule out diagnoses.

^¶¶^ Includes all immunocompromising conditions listed above plus chronic heart disease (including congestive heart failure and cardiomyopathies, excluding hypertension), chronic lung disease (including chronic obstructive pulmonary disease, emphysema, and asthma), and diabetes mellitus.

Claims for pneumococcal vaccination by September 18, 2016 among beneficiaries aged ≥65 years also varied by state of residence (Table). Claims for PPSV23 ranged from 25.4% in Alaska to 52.3% in Wisconsin, claims for PCV13 ranged from 20.7% in Mississippi to 53.9% in Wisconsin, and claims for both vaccines ranged from 9.4% in Alaska to 34.9% in Wisconsin.

Monthly claims for PCV13 among beneficiaries aged ≥65 years after publication of the September 2014 recommendation increased from 0.9% in September 2014 to 22.9% in December 2015 (Figure 2). The steepest monthly increase (4 percentage points) occurred from September 2015 to October 2015. During January–September 2016, the average monthly increase was 0.71 percentage points.

**FIGURE 2:**
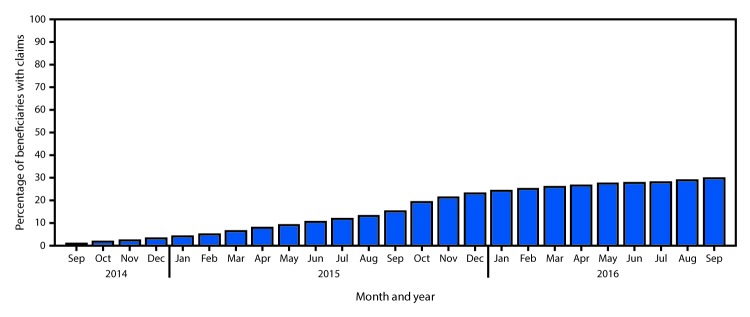
Percentage of Medicare beneficiaries aged ≥65 years with claims submitted for 13-valent pneumococcal conjugate vaccine (PCV-13), by month* — United States, September 2014–September 2016^†^ * Percentage of beneficiaries with at least one claim for PCV13 before the end of the month of interest. Denominator each month includes beneficiaries continuously enrolled in Medicare Parts A and B for at least 12 months before and including the month of interest. ^†^ The Advisory Committee on Immunization Practices recommendation for the routine use of PCV13 for adults aged ≥65 years was published September 19, 2014.

## Discussion

PPSV23 has been demonstrated effective in preventing invasive pneumococcal disease (IPD) in adults. However, approximately 20%–25% of IPD cases and 10% of community-acquired pneumonia cases in adults aged ≥65 years are caused by serotypes unique to PCV13. Broader protection against pneumococcal disease is expected through use of both PCV13 and PPSV23 in series (*1*). In 2014, when ACIP recommended routine use of PCV13 in series with PPSV23 among adults aged ≥65 years, the addition of PCV13 was estimated to prevent 230 cases of IPD and approximately 12,000 cases of community-acquired pneumonia over the lifetime of a single cohort of persons aged 65 years in the United States (*1*). Two years after the ACIP recommendation for routine use of PCV13 in series with PPSV23 in adults aged ≥65 years, claims for PCV13 rose steadily, to 31.5% in September 2016. However, the expected benefits of PCV13 use in terms of cases of IPD and pneumonia prevented were estimated in a setting of 60% coverage (*4*). Claims for PPSV23 were also persistently low, despite a long-standing recommendation for PPSV23 use in this population (*5*). The steepest increase in PCV13 uptake coincided with the beginning of the 2015–16 influenza season, suggesting that older adults might be receiving pneumococcal vaccination when they go to their providers for influenza vaccination. Implementation of the standards for adult immunization practice (*2*) could help improve the initiation and completion of the pneumococcal vaccination series among adults aged ≥65 years to reduce the incidence of pneumonia and invasive pneumococcal disease among these persons.

Implementation of PCV13 and PPSV23 vaccination has not been equal across subgroups of adults aged ≥65 years. White beneficiaries were more likely to have claims for either type of vaccine than were beneficiaries of other racial/ethnic groups, especially Hispanics and blacks. Differences in coverage with pneumococcal vaccine, as well as other vaccines, among older adults by race/ethnicity are well documented, and might be attributable to differences in attitudes toward vaccination and concerns about vaccination safety, in provider recommendation for vaccination, and in quality of care received by different racial/ethnic groups (*6*,*7*). Although PPSV23 and PCV13 are now routinely recommended for all adults aged ≥65 years, beneficiaries aged ≥65 years with chronic or immunocompromising medical conditions were more likely to have been vaccinated with both vaccines than were beneficiaries without such conditions. This higher percentage might be attributable to several factors: beneficiaries with chronic or immunocompromising medical conditions having more frequent provider contacts, and thus more opportunities for vaccination; providers being more aware of vaccination needs for persons with complicated medical conditions; and patients with chronic or immunocompromising conditions being more aware of the need for pneumococcal vaccination. Vaccination with both types of pneumococcal vaccine also varied by state, as has been previously reported for PPSV23 (*7*). State variation in vaccination coverage has been attributed to differences in health care delivery infrastructure and vaccination programs, as well as differences in population characteristics between states (*7*).

The findings in this report are subject to at least five limitations related to the use of Medicare claims data as a proxy for estimating vaccination coverage. First, the percentage of beneficiaries in this study population with claims for pneumococcal vaccination might not be representative of pneumococcal vaccination coverage among all adults aged ≥65 years in the United States. The percentage with claims for any pneumococcal vaccine in this study (56.4%) differs from the 63.5% reported coverage with any pneumococcal vaccine among adults aged ≥65 years from the nationally representative 2015 National Health Interview Survey (*8*). Second, exclusion from the current analysis of beneficiaries enrolled in Medicare Part C (Medicare is not billed separately for vaccinations for beneficiaries enrolled in Part C health plans) might have contributed to over- or underrepresentation of vaccinated persons in the study population. In 2016, 33% of Medicare beneficiaries were enrolled in Part C, with enrollment by state ranging from 1% to 58%^§^ (*9*). Third, the CMS database does not include claims for vaccinations administered before 1999. Whereas not having information on pneumococcal vaccination claims before 1999 would not affect estimates for PCV13 vaccination, the percentage of persons vaccinated with PPSV23 could be underestimated, particularly among older beneficiaries who reached age 65 years before 1999 and might have been vaccinated with PPSV23 after its licensure for use in the United States in 1983. Fourth, doses administered during hospitalization might not be captured if claims for the hospital stay were bundled. Finally, race/ethnicity of Hispanic beneficiaries and those of races other than white or black could potentially be misclassified because of the change in categorization of race/ethnicity information collected by the Social Security Administration in 1980 (*10*).

Despite these limitations, the use of Medicare claims data are an efficient mechanism to monitor the acceptance of PCV13 and PPSV23 among adults aged ≥65 years. The ACIP will reevaluate the recommendation for routine use of PCV13 in adults aged ≥65 years in 2018 and revise as needed (*1*). Timely assessment of PCV13 uptake and completion of the pneumococcal vaccination series with PCV13 and PPSV23 are necessary to evaluate prevention of pneumococcal pneumonia and invasive pneumococcal disease by vaccination. To reduce the incidence of pneumococcal disease, providers should ensure that older adults initiate and complete the recommended pneumococcal vaccination series.

**Summary**

**What is already known about this topic?**

On September 19, 2014, CDC published a recommendation of the Advisory Committee on Immunization Practices (ACIP) for routine use of 13-valent pneumococcal conjugate vaccine (PCV13) among adults aged ≥65 years, to be used in series with 23-valent pneumococcal polysaccharide vaccine (PPSV23). ACIP will reevaluate the recommendation for routine use of PCV13 in adults aged ≥65 years in 2018 and revise as needed.

**What is added by this report?**

Among Medicare beneficiaries (Parts A and B) aged ≥65 years, 43.2% had received ≥1 dose of PPSV23, 31.5% had received ≥1 dose of PCV13, and 18.3% had received both PCV13 and PPSV23 by September 18, 2016. Receipt of either type of pneumococcal vaccine was highest among beneficiaries who were older, white, or with chronic and immunocompromising medical conditions. Claims for PPSV23 vaccination were persistently low despite long-standing recommendations for its use among adults aged >65 years.

**What are the implications for public health practice?**

Initiation and completion of the pneumococcal vaccination series among adults aged ≥65 years can be improved by implementation of the standards for adult immunization practice. Estimates of vaccination with PCV13 and PPSV23 in adults aged ≥65 years are important factors in the consideration of the revision of the recommendation for routine use of PCV13.

## Footnotes

* Analysis includes only Medicare beneficiaries in fee-for-service plans (Medicare Parts A and B). Beneficiaries receiving Medicare services through Medicare Advantage or other health plan (Medicare Part C) are excluded. Under Part C health plans, Medicare is not billed separately for vaccinations.

† Beneficiaries identified as Hispanic or Latino might be of any race. Beneficiaries identified as black, white, Asian, American Indian/Alaskan Native, or other race are non-Hispanic. “Other” includes persons of multiple race. Race/ethnicity information for Medicare beneficiaries was historically obtained from the Social Security Administration’s master beneficiary record. Before 1980, the Social Security application form only allowed classification of race into White, Black, and Other. Since 1980, the categories have been expanded to White (non-Hispanic); Black (non-Hispanic); Hispanic; Asian, Asian American, or Pacific Islander; American Indian or Alaska Native; and Unknown. The Health Care Financing Administration (now Centers for Medicare & Medicaid Services) conducted surveys of beneficiaries in attempts to better classify race/ethnicity of those enrolled before 1980. However, misclassification of race/ethnicity among beneficiaries included in the current analysis might remain, particularly those of Hispanic ethnicity and races other than white or black.

§ Although data are not available regarding differences in vaccination coverage between beneficiaries enrolled in Parts A and B versus Part C, if differences do exist, some of the variation in claims rates by state could be attributable to the large variation in the percentage of beneficiaries enrolled in Part C across states. The national estimate based on beneficiaries enrolled in Parts A and B could be biased even if coverage among Part C beneficiaries was similar, if residents of states with higher coverage are underrepresented because of a higher percentage of enrollment in Part C compared with residents of states with lower vaccination coverage.
