# Erratum: Vol. 66, No. 27

**DOI:** 10.15585/mmwr.mm6632a8

**Published:** 2017-08-18

In the report “Pneumococcal Vaccination Among Medicare Beneficiaries Occurring After the Advisory Committee on Immunization Practices Recommendation for Routine Use Of 13-Valent Pneumococcal Conjugate Vaccine and 23-Valent Pneumococcal Polysaccharide Vaccine for Adults Aged ≥65 Years,” on page 732, the second sentence of the discussion should have read “However, approximately 20%–25% of IPD cases and 10% of community-acquired pneumonia cases in adults aged ≥65 years are caused by **PCV13 serotypes**.”

